# HES1 Protein Modulates Human Papillomavirus–Mediated Carcinoma of the Uterine Cervix

**DOI:** 10.1200/JGO.18.00141

**Published:** 2019-01-07

**Authors:** Richa Tripathi, Gayatri Rath, Vishwas Sharma, Showket Hussain, Shashi Sharma, Mausumi Bharadwaj, Ravi Mehrotra

**Affiliations:** ^1^ Indian Council of Medical Research–National Institute of Cancer Prevention and Research, Noida, India; ^2^ Vardhman Mahavir Medical College and Safdarjung Hospital, New Delhi, India; ^3^ Society for Life Sciences and Human Health, Allahabad, India

## Abstract

**PURPOSE:**

Cervical cancer (CC) is the most common cancer affecting women worldwide. Human papillomavirus (HPV) infection is a major contributing factor for the development of CC. The development of CC occurs progressively from precancer stages to cancerous stages (ie, invasive squamous cell carcinoma [ISCC] and adenocarcinoma [ADC]). ADC is a rare form of CC that develops from the mucinous endocervical epithelium. It is believed that the downstream targets of Notch signaling contribute to the etiology of CC. One such target is HES1, whose role in the modulation of ADC is unknown. The purpose of this study is to determine the role of HES1 protein in HPV-associated ADC subtype of CC and also to compare its expression in histologic subtypes of precancer and ISCC.

**PATIENTS AND METHODS:**

A total of 148 patients (30 with precancers, 98 with ISCC, and 20 with ADC) and 40 normal control participants were analyzed for the expression of HES1 via immunohistochemistry, with results validated by immunoblotting.

**RESULTS:**

The comparison between HPV-16 and HES1 expression was significant in precancer (cervical intraepithelial neoplasia grades 1 to 3; *P* = .013), ISCC (International Federation of Gynecology and Obstetrics stages I to IV; *P* = .001), and ADC (*P* = .007). An overall significant mean difference was observed between HES1, JAG1, and Notch-3 proteins in precancer (*P* = .001), ISCC (*P* = .001), and ADC (*P* = .001). Pairwise comparisons between HES1 and JAG1 and HES1 and Notch-3 were also found to be significant.

**CONCLUSION:**

This study showed that among all HPV-16–positive precancers, the major HES1 positivity signal arises from cervical intraepithelial neoplasia grades 2 and 3 that develops into ISCC. Moreover, HPV-16–positive ADC also showed an association with HES1. The HES1, JAG1, and Notch-3 proteins showed their synergistic role in modulating HPV associated ADC along with histologic subtypes of precancer and ISCC of CC.

## INTRODUCTION

Hairy and enhancer of split homolog-1 (HES1) is a downstream effector target for mammalian Notch signaling pathway.^1^ The *Hes* family comprises seven members, *Hes1* to *Hes7,* all of which are structurally conserved and broadly classified into two groups based on their regulation by *Notch*.^1^ The active form of Notch activates the promoter of *Hes1*, which affects its expression and is believed to inhibit cell differentiation and promote the survival of stem cells.^2,3^ The overactivation of HES1 is believed to influence the balance between cell proliferation and differentiation.^4^ Liu et al^5^ demonstrated a role of HES1 in cancer stem cells, metastasis, and cell fate. It has also been shown that HES1 promotes cell proliferation in cervical cancer (CC) and thus may be involved in carcinogenesis of the cervix and progression of CC.^1,6^

CC ranks fourth among female-related cancers worldwide, with a reported incidence of approximately 528,000 new cancers every year.^7^ In India, its incidence rate is 122,844 cancers per year, and CC is reported to be the second most common cancer affecting women age 15 to 44 years.^8^ Infection with high-risk subtypes of human papillomavirus (HPV; types 16 and 18) plays a preponderant role in the development of precancer of the uterine cervix (UC) and the majority of CC subtypes. High-risk HPV encodes multifunctional growth-promoting E6 and E7 oncoproteins that bind and inactivate tumor suppressor genes. In HPV-infected cells, a complex network of protein interactions has been reported with other key signaling pathways such as Notch, TGFβ/SMAD, and WNT/β-catenin.^9^ However, the altered molecular mechanisms responsible for activation of various pathways leading to CC remain unknown.

Histologically, precancer of UC is classified by the Bethesda System^10^ into squamous intraepithelial lesion subtypes or cervical intraepithelial neoplasia (CIN; grades 1 to 3). Low-grade squamous intraepithelial lesions include mild dysplasia or CIN 1; high-grade squamous intraepithelial lesions include moderate dysplasia or CIN 2; and severe dysplasia includes carcinoma in situ or CIN 3.^10^ Similarly, cancer of UC is divided into invasive squamous cell carcinoma (ISCC) and adenocarcinoma (ADC).^11^ CC is classified by the International Federation of Gynecology and Obstetrics (FIGO) system according to the clinical and/or pathologic spread of disease.^12^ ISCC is the most common type of CC, composing 85% to 90% of CCs in India. ADC, a rare subtype, develops from the mucinous endocervical epithelium (mucus-producing gland cells)^13^ and is classified as either ADC in situ (precursor lesion) or invasive ADC. It is generally difficult to diagnose ADC in situ because pathologists cannot use the basement membrane for the measurement of invasion. ADC of UC has chemotherapy- and radiotherapy-resistant properties; therefore, the response to bevacizumab plus paclitaxel and cisplatin therapy, which is a gold standard therapy for ISCC, is not significant in ADC.^14^ Unlike squamous cell carcinoma, the prevalence of ADC is increasing worldwide at an alarming rate.^14^ However, in India, it composes 10% to 15% of CCs.^11^ Moreover, in North India, it is rare for patients with ADC to visit hospitals; therefore, treating these patients is a challenging task. In addition, the majority of patients with CC report to hospitals at late stages, and therefore, diagnosis of patients with CC occurs at late stages, underscoring the need to improve early diagnosis and treatment via targeted therapies among women. To do this, we must understand the molecular mechanisms of the disease. To our knowledge, there is no study available to date regarding the molecular mechanisms of ADC of CC (Data Supplement). However, with respect to precancer and ISCC of UC, only four studies are available suggesting that HES1 may directly drive tumorigenesis inducing neoplastic transformation.^1,15-17^ Therefore, this study aims to fill the research gap by studying the role of HES1 expression in HPV-associated ADC, as well as in precancer and ISCC of CC. This will help us to clarify the role of HES1 in the progression of this neoplastic disease.

## PATIENTS AND METHODS

### Study Design and Participants

A total of 148 patients (30 with precancers, 98 with ISCC, and 20 with ADCs) and 40 normal control participants (ultraviolet prolapse, nonneoplastic tissues) were enrolled from the Department of Obstetrics and Gynecology of the Safdarjang Hospital in New Delhi, India. Approval was provided by the Research and Ethics Committee of the National Institute of Cancer Prevention and Research–Indian Council of Medical Research in Noida, India, and Vardhman Mahavir Medical College and Safdarjang Hospital in New Delhi, India. All untreated individuals enrolled onto the study with no family history of CC provided written informed consent. Patients with precancers included 19 with CIN 1, three with CIN 2, and eight with CIN 3. The clinical staging of tumors was confirmed according to the FIGO classification criteria of tumor staging along with its respective histopathologic grade by two independent pathologists.^12,18^ Among ISCCs (n = 98), 33 were FIGO stage I or II and 65 were FIGO stage III or IV. All of the collected ADC samples were invasive ADC and pathologic grade 2 or 3 and FIGO stage III or IV. All patients were diagnosed by colposcopic biopsy; however, ADCs were more difficult to diagnose than ISCCs. In addition, all biopsy samples were used for molecular investigations.

All data generated or analyzed during this study are included in this published article and its Data Supplement. The raw data files used in the article can be provided upon request.

### Immunohistochemistry

Formalin-fixed paraffin-embedded specimens were processed for fine sections (5 μm) and were mounted on slides coated with poly-l-lysine (Sigma, St Louis, MO), followed by conventional hematoxylin and eosin staining for histologic assessment and immunohistochemistry (IHC). Citrate buffer (pH 6.0) was used for antigen retrieval treatment in the microwave and incubated overnight at 4°C with the primary rabbit polyclonal antibody of HES1 (ab87395; Abcam, Cambridge, United Kingdom) at a dilution of 1:250 respectively. An Envision System peroxidase kit (DAKO, Carpinteria, CA) was used for staining. Detailed methodology has been described earlier.^19^

### Scoring of IHC

IHC results for HES1 protein followed the scoring criteria provided by Tripathi et al.^19^ A total score was obtained by adding the percent positivity and intensity scores.

### Immunoblotting

Immunoblotting from all cervical tissues was done according to a previous study from our laboratory.^19^ Polyclonal antihuman antibodies of HES1, β-actin (rabbit monoclonal β-actin antibody (1:2,000; Abcam), and diluted secondary antibody horseradish peroxidase–conjugated rabbit anti-IgG (Abcam) were used in this study. The expression levels of HES1 in tumor tissues were quantitated and compared with their expression levels in normal tissues and were evaluated by densitometry using Alpha Digidoc version 4.1.0 (Alpha Innotech, San Leandro, CA), as described in our previous report.^19^

### Statistical Analysis

Patients with precancer (CIN 1, CIN 2, or CIN 3) were categorized into two groups (CIN 1 *v* CIN 2 and CIN 3) as a result of the small number of patients with CIN 2 (n = 3). Similarly, ISCC patients were also categorized into the following two groups: FIGO stage I and II and FIGO stage III and IV. Patients with ADC were not categorized separately because of their limited numbers and their higher pathologic grade (2 and 3) and FIGO stage (III and IV). The analysis of HES1 protein expression in patients with precancer, ISCC, and ADC, along with their correlation with HPV subtypes was evaluated using the χ^2^ test. The nonparametric Kruskal-Wallis test was applied for comparison of HES1, JAG1, and Notch-3 because the data were not normally distributed. The overall mean differences between these proteins and pairwise comparisons were performed using the Mann-Whitney *U* test between groups, as follows: HES1 and JAG1, HES1 and Notch-3, and JAG1 and Notch-3. SPSS (version 20; SPSS, Chicago, IL) statistical software was used for all analysis. *P* ≤ .05 was considered significant.

## RESULTS

### Correlation of HES1 Expression in HPV-Infected Precancers, ISCCs, and ADCs

#### Precancer.

The comparison between HPV-negative or -positive status and HES1 expression was found to be nonsignificant (*P* = .154) in CIN 1 (Table 1). However, in patients with CIN 2 and 3, it was found to be significant (*P* = .011). In addition, the comparison between HPV-negative or HPV-positive status and HES1 expression in all patients with CIN was found to be significant (*P* = .013). Among all patients with HPV-16–positive precancer, HES1 positivity was observed in 83.3% (20 of 24 patients; *P* = .013). This implies that the major HES1 positivity signal in patients with HPV-16–positive precancer arises from CIN 2 and 3. However, none of the patients with precancer were found to be infected with HPV-18; therefore, this comparison was not done.

**TABLE 1:**
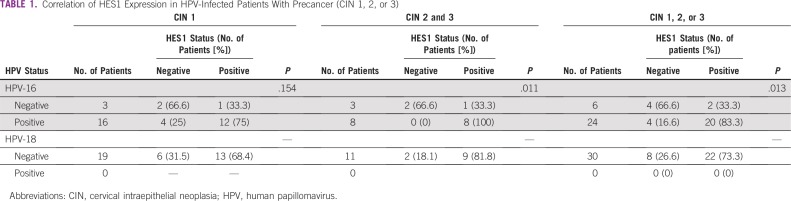
Correlation of HES1 Expression in HPV-Infected Patients With Precancer (CIN 1, 2, or 3)

#### ISCC.

The comparison between HPV-negative or -positive status and HES1 expression in less invasive ISCC (FIGO stage I or II) was found to be significant (*P* = .034). Similarly, this comparison was also found to be significant in patients with highly invasive ISCC (FIGO stage III or IV; *P* = .023) and in all patients with ISCC (FIGO stage I to IV; *P* = .001; Table 2). This signifies that the majority of HES1 expression came from HPV-16–positive CIN 2 and 3 and was also found to be intensified in all ISCCs.

**TABLE 2:**
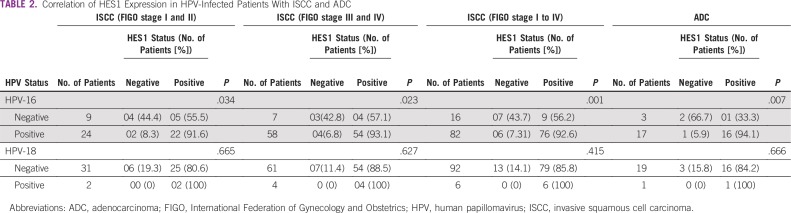
Correlation of HES1 Expression in HPV-Infected Patients With ISCC and ADC

Therefore, in patients with ISCC, HPV-16 was found to be significantly linked with HES1 expression in all subgroups. However, we did not find a significant association between HPV-18 and HES1, which could be a result of the small number of HPV-18–positive patients.

#### ADC.

A significant association between HES1 (*P* = .007) and HPV-16–positive ADC was observed (Table 2). Only 1 of 20 patients was infected with HPV-18 and found to be HES1 positive (*P* = .666; Table 2).

**FIG 1:**
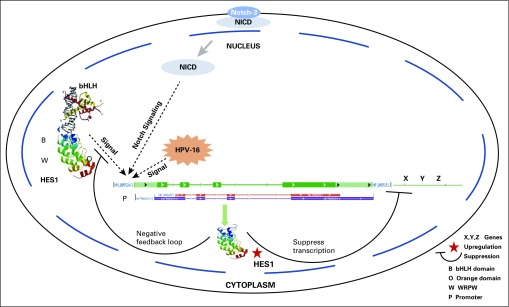
Overview of HES1 regulation in cervical cancer. HES1-bHLH protein complex, Notch-3, and human papillomavirus (HPV) subtype 16 signals induce the transcription and translation of HES1 protein, resulting in the regulation of its own expression through a negative feedback loop. Subsequently, HES1 also suppresses the transcription of various genes involved in cell proliferation and differentiation. NICD, Notch intracellular domain.

The overall results suggest the hypothesis that the altered HES1 plays a regulating role in precancer, ISCC, and ADC by synergizing with HPV-16 as the aggressiveness of the tumor increases. The increased expression level of this protein in the nucleus suggests its association with bHLH protein^20^ and regulation of its own expression through a negative feedback loop and, subsequently, suppression of the transcription of various genes that influence cell proliferation and differentiation (Fig 1).

### Expression Profile of HES1 Protein in Precancer, ISCC, and ADC Lesions of the Human Cervix by IHC

**FIG 2:**
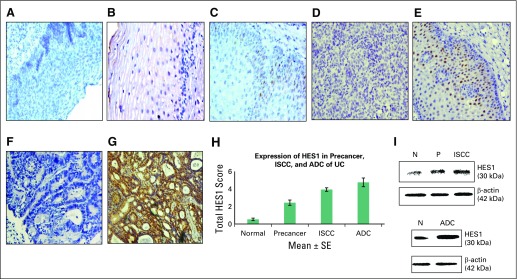
Expression analysis of HES1 in normal, precancer, invasive squamous cell carcinoma (ISCC), and adenocarcinoma (ADC) tissues of uterine cervix (UC). Immunohistochemical analysis showed (A) negative control in normal tissue (magnification, ×200); (B) no nuclear expression of HES1 in normal cervix (×200); (C) mild nuclear expression of HES1 in precancer (×200); (D) negative control in ISCC tissue (×200); (E) moderate nuclear expression of HES1 in ISCC (×200); (F) negative control in ADC (×200); and (G) intense nuclear localization of HES1 in ADC (×200). (H) Bar graph showing expression of HES1 in normal, precancer, ISCC, and ADC samples. (I) Western blots depict the expression of HES1 in normal (N), precancer (P), ISCC, and ADC tissues.

The expression profile (Fig 2A to Fig 2G) and total expression scores (Table 3) of HES1 in the nucleus were determined in normal, precancer, ISCC, and ADC tissues. An approximate four-fold increase (mean ± SE, 2.43 ± 0.30; *P* < .001) in nuclear HES1 expression was identified in normal versus precancer tissue, a seven-fold increase (mean ± SE, 3.76 ± 0.21; *P* < .001) was observed in normal versus ISCC tissue, and an eight-fold increase (mean ± SE, 4.75 ± 0.50; *P* < .001) was observed in normal versus ADC tissue. Among patients with precancer (CIN 1 and CIN 2 and 3), HES1 expression is provided in the Data Supplement. The expression of HES1 in two ISCC subcategories is also provided in the Data Supplement. Figure 2H shows total IHC HES1 expression scores in normal cervix, precancer, ISCC, and ADC biopsies. Hence, an overall gradual increase in HES1 expression was observed in moving from precancer to ISCC and ADC.

**TABLE 3:**
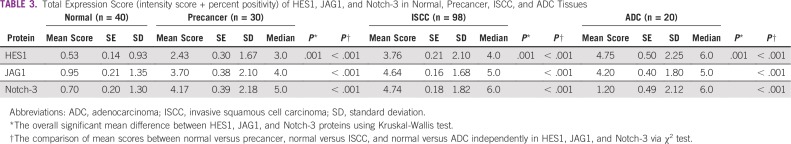
Total Expression Score (intensity score + percent positivity) of HES1, JAG1, and Notch-3 in Normal, Precancer, ISCC, and ADC Tissues

### Combined Impact of HES1 and JAG1 or HES1 and Notch-3 Expression on Precancer, ISCC, and ADC

We analyzed the combined effect of HES1 and JAG1 and of HES1 and Notch-3 on precancers, ISCC, and ADC (Table 3 and Data Supplement). For this purpose, we used the JAG1 data from precancers, ISCC, and ADC from our previous report^21^; Notch-3 precancer and ISCC data from our previously published study^19^; and ADC data from our unpublished records (IHC figures available upon request). An overall significant mean difference was observed between HES1, JAG1, and Notch-3 proteins in precancer (*P* = .001), ISCC (*P* = .001), and ADC (*P* = .001).

Pairwise comparisons between HES1 and JAG1 and between HES1 and Notch-3 were also found to be statistically significant in precancer, ISCC, and ADC. This fact implies that all of these proteins should be studied concomitantly in the future to understand the complex cascade of events modulating precancer, ISCC, and ADC.

### Receiver Operating Characteristic Curve Analysis: Potential Determination of HES1 Expression to Distinguish Precancer, ISCC, and ADC From Normal Cervical Tissue

**FIG 3:**
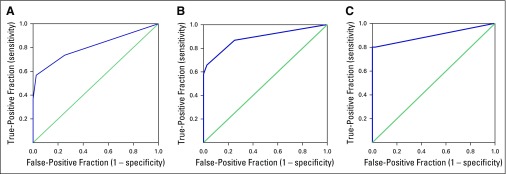
Receiver operating characteristic curve of nuclear HES1 in (A) normal versus precancer samples; (B) normal versus invasive squamous cell carcinoma samples; and (C) normal versus adenocarcinoma samples. The *y*-axis represents true-positive fraction, whereas the *x*-axis shows false-positive fraction.

The area under the curve (AUC) values for nuclear HES1 in precancer (AUC, 0.80; *P* < .001), ISCC (AUC, 0.88; *P* < .001), and ADC (AUC, 0.90; *P* < .001) were determined (Fig 3 and Data Supplement). The sensitivity and specificity for nuclear HES1 in precancer, ISCC, and ADC were 73.30% and 89.80%, 86.7% and 75%, and 85% and 75%, respectively. High sensitivity and specificity of HES1 in precancer, ISCC, and ADC support the clinical utility of HES1 for early detection and progression of CC.

### Clinicopathologic Parameters of ISCC and IHC Expression of HES1

The clinicopathologic parameters of ISCC showed association with HES1 expression as follows (Data Supplement). HES1 was found to be associated with tobacco (88.9%; *P* = .04), tumor size (90.2%; *P* < .001), tumor vaginal involvement (89.2%; *P* < .001), lymph node metastasis (87.7%; *P* < .002), and FIGO stage (89.2%; *P* < .001). These results imply the pathogenic aggressiveness of CC when associated with HES1.

### Immunoblotting

A gradual increase in the expression of HES1 (30 kDa) identified via immunoblotting in precancer, ISCC, and ADC samples (Fig 2) validates the findings of IHC.

## DISCUSSION

A growing understanding of the complex signaling pathways that underlie progression of a tumor is driving the development of new therapeutic targets at specific molecular events. Currently, there is no targeted therapy available for CC because the molecular mechanisms underlying CC development and progression remain poorly defined. Evidence suggests that HPV infection alone is inadequate to induce malignant changes and that altered signaling pathways are also important for the development of CC. It was hypothesized that activated Notch-3 and JAG1 interact synergistically with HPV oncoproteins E6 and E7 and induce the Notch signaling pathway, promoting cell proliferation and tumorigenesis.^19,21^ *HES1* was reported to be a Notch-targeted gene by showing suppression of Notch signaling–mediated gene expression by DN-MAML expression, leading to decrease of HES1.^22^

This study compared HES1 expression in HPV-negative and -positive patients with precancer (CIN 1 *v* CIN 2 and 3), ISCC (FIGO stage I and II *v* III and IV), and ADC and suggests that, among all patients HPV-16–positive precancers, the major HES1 positivity signal arises from CIN 2 and 3 precancers that develop into ISCC. Moreover, HPV-16–positive patients with invasive ADC also showed an association with HES1.

The expression of HES1 in precancer, ISCC, and ADC of UC was found to be significantly upregulated progressively as compared with normal cervix tissue. This implies an increasing trend of HES1 expression from HPV-16–positive precancer to ISCC and ADC. This hypothesis was supported by Liu et al^6^ in 2010; however, it needs to be explored further. Our results are in concordance with previous studies in which the upregulation of HES1 both at the RNA and protein levels was identified but only in precancer and ISCC.^1,15-17^ Briefly, Liu et al^1^ found higher expression of HES1 only in CIN lesions, and no difference was found in ISCC. However, Ramdass et al^16^ observed elevated expression in ISCC but only through IHC. The present study extends the previous findings by adding the knowledge of HES1 upregulated expression in HPV-infected ADC (rare subtype of CC) and compared the expression in HPV-associated precancer, HPV-associated ISCC, and HPV-associated ADC. In addition, we have extended our work with respect to validation of IHC results by Western blotting. Rong et al^17^ also investigated HES1 at the RNA level by quantitative reverse transcription polymerase chain reaction and observed similar increased expression levels in CC tissues.

A combined impact and significant mean difference of HES1 and JAG1 and of HES1 and Notch-3 were observed in precancer, ISCC, and ADC. Hence, HES1, JAG1, and Notch-3 are interrelated with each other. This confirms that HES1 is an effector target of triggered activation of JAG1-induced Notch signaling, which could be of potential diagnostic utility in early- and late-stage patients with CC. Hence, the previously observed activated Notch pathway, in turn, alters HES1 progressively in the modulation of CC cell proliferation.

High sensitivity and specificity of nuclear HES1 in precancer, ISCC, and ADC strongly support its clinical significance for early detection, progression, and monitoring of patients with cervical dysplasia. 

An association between clinicopathologic parameters of patients with ISCC and nuclear HES1 was observed with respect to tumor size, tumor vaginal invasion, FIGO stage, and lymph node metastasis, suggesting the contribution of HES1 to progression, aggressiveness, and invasion of tumor, thereby showing its potential clinical utility as a candidate predictive marker for CC.

To improve patient care with targeted therapies that lead to more accuracy and specificity, the combined analysis of HES1, JAG1, and Notch-3 in ADC is required. This pilot study on ADC is a step toward understanding the role of HES1 in the HPV-16–induced Notch signaling pathway in the rare ADC subtype of CC. Hence, this report may be a torchbearer for researchers and clinicians to understand the involvement of Notch signaling in regulating molecular alterations of HES1 in HPV-associated precancer, ISCC, and ADC. Using HES1 as a targeted therapy in combination with other chemotherapeutics drugs may be a novel approach to abolish or manage this deadly neoplastic phenotype to improve survival times in patients with ISCC and ADC. However, future studies need to be done in a large cohort of patients with ADC (both ADC in situ and invasive ADC) to confirm our findings. This study may help in designing future studies, understanding the molecular mechanism modulating CC, and targeting HES1 via molecular therapeutics.
